# Comparative and Functional Screening of Three Species Traditionally used as Antidepressants: *Valeriana officinalis* L., *Valeriana jatamansi* Jones ex Roxb. and *Nardostachys jatamansi* (D.Don) DC.

**DOI:** 10.3390/plants9080994

**Published:** 2020-08-05

**Authors:** Laura Cornara, Gabriele Ambu, Domenico Trombetta, Marcella Denaro, Susanna Alloisio, Jessica Frigerio, Massimo Labra, Govinda Ghimire, Marco Valussi, Antonella Smeriglio

**Affiliations:** 1 Department of Earth, Environment and Life Sciences, University of Genova, 16132 Genova, Italy; laura.cornara@unige.it (L.C.); frategabriele@libero.it (G.A.); 2 Department of Chemical, Biological, Pharmaceutical and Environmental Sciences, University of Messina, Via Giovanni Palatucci, 98168 Messina, Italy; dtrombetta@unime.it (D.T.); mdenaro@unime.it (M.D.); 3 ETT Spa, via Sestri 37, 16154 Genova, Italy; susanna.alloisio@ettsolutions.com; 4 Institute of Biophysics-CNR, 16149 Genova, Italy; 5 FEM2 Ambiente Srl, Piazza della Scienza 2, 20126 Milan, Italy; jessica.frigerio@fem2ambiente.com; 6 Department of Biotechnology and Bioscience, University of Milano-Bicocca, Piazza della Scienza 2, 20126 Milan, Italy; massimo.labra@unimib.it; 7 Nepal Herbs and Herbal Products Association, Kathmandu 44600, Nepal; ghimiregovinda31@gmail.com; 8 European Herbal and Traditional Medicine Practitioners Association (EHTPA), Norwich 13815, UK; marco@gadoi.it

**Keywords:** Caprifoliaceae, essential oil, acetylcholinesterase, neuroactive effects, MEA analyses, DNA barcoding, micromorphology, botanicals authentication

## Abstract

The essential oils (EOs) of three Caprifoliaceae species, the Eurasiatic *Valeriana officinalis* (Vo), the Himalayan *Valeriana jatamansi* (Vj) and *Nardostachys jatamansi* (Nj), are traditionally used to treat neurological disorders. Roots/rhizomes micromorphology, DNA barcoding and EOs phytochemical characterization were carried out, while biological effects on the nervous system were assessed by acetylcholinesterase (AChE) inhibitory activity and microelectrode arrays (MEA). Nj showed the highest inhibitory activity on AChE (IC_50_ 67.15 μg/mL) followed by Vo (IC_50_ 127.30 μg/mL) and Vj (IC_50_ 246.84 μg/mL). MEA analyses on rat cortical neurons, carried out by recording mean firing rate (MFR) and mean bursting rate (MBR), revealed stronger inhibition by Nj (IC_50_ 18.8 and 11.1 μg/mL) and Vo (16.5 and 22.5 μg/mL), compared with Vj (68.5 and 89.3 μg/mL). These results could be related to different EO compositions, since sesquiterpenes and monoterpenes significantly contribute to the observed effects, but the presence of oxygenated compounds such as aldehydes and ketones is a discriminating factor in determining the order of potency. Our multidisciplinary approach represents an important tool to avoid the adulteration of herbal drugs and permits the evaluation of the effectiveness of EOs that could be used for a wide range of therapeutic applications.

## 1. Introduction

Medicinal and aromatic plants have played a fundamental role, from ancient times to today, because they have been used for many therapeutic purposes all over the world [1,2]. Some of these plants contain chemicals with sedative, stimulating and hallucinogenic effects, and are often used in traditional medicine for the treatment of CNS disorders [3,4]. Many studies have been focused on the therapeutic potential of plant-derived psychoactive compounds; however, only a few mechanisms of action have been clarified to date [5,6].

Among the most popular herbal medicines used for their sedative and anxiolytic effects, different species belonging to the “Valerianaceae” family (actually, Caprifoliaceae), including more than 200 species widespread in Europe, North America and Asia. Many of them are well known in Ayurvedic, Chinese and Tibetan medicine as a part of different preparations indicated to treat nervous and emotional disorders, insomnia, epilepsy, madness, and various human ailments too [2,4,7,8,9,10,11]. In the present study we investigated three species of Caprifoliaceae, to which previous studies and literature have attributed psychoactive value, i.e., *Valeriana officinalis* L. (Vo), *Valeriana jatamansi* Jones ex Roxb. (Vj) and *Nardostachys jatamansi* (D.Don) DC. (Nj) [12,13,14]. The roots of Vo and Nj, because of high commerce, are often fraudulently adulterated with other species [15]. In addition, confusion about the botanical nomenclature between Nj and Vj frequently has been observed, since both the species are known with the same vernacular names, and the authentication of herbal material is made more difficult by the use of dried root/rhizome samples [16].

Vo is a perennial herb common in North America, Europe, and Asia. Its rhizome is short and stoloniferous, and the roots are fibrous and have a characteristic smell [12]. Studies on Vo root extract have revealed many chemical compounds with a broad spectrum of biological activities on the CNS such as sesquiterpenes (valerenic acid and derivatives) and valepotriates, flavonoids, triterpenes, lignans, alkaloids, amino acids such as c-aminobutyric acid and γ- aminobutyric acid (GABA) as well as quinolinic acid with antioxidant and lipid peroxidation-decreasing effects [12,17]. In traditional medicine, Vo root has been used mainly for its sedative, hypnotic and anxiolytic properties in the treatment of insomnia, anxiety, epilepsy, and neurodegenerative diseases such as Parkinson’s disease and Alzheimer’s disease [18,19].

Vj is a small perennial herb distributed in the Himalaya region at an altitude of 1500–3000 m. The rhizome is thick and horizontal, whereas the descending roots are fibrous. Rhizomes and roots contain important compounds to which the therapeutic properties are attributed: valepotriates, sesquiterpenoids, valeriananoids, jatamanins, lignans, cryptomeridiol, maaliol, xanthorrhizzol, patchouli alcohol and others [13,20]. In Indian systems of medicine, Vj roots have long been used to treat various diseases and as a substitute for Vo in the treatment of nervous disorders [21]. In particular, the sedative properties are attributed to the presence of the valepotriates [22].

Nj is a small, perennial herb distributed in Himalaya at an altitude of about 3000–5000 m. Its rhizome is woody and is covered with brown fibrous remains of the petioles of the radical leaves, while the roots penetrate deep in the soil. Nj has been used since ancient times by traditional medicine systems such as Ayurveda, Siddha and Unani to treat a wide range of ailments [16]. Rhizomes and roots of Nj are mainly used for various neurological disorders such as hysteria, epilepsy, and mental weakness [23]. Rhizomes and roots contain many bioactive compounds such as sesquiterpene, coumarins, lignans, neolignans, alkaloids, among which the most active constituents are sesquiterpenes and coumarins [24].

Valerian essential oils (EOs) and extracts are used in flavor, pharmaceutical and fragrance industries and about 30 products are commercially available [7,25], for example, Valmane^®^ extract from *V. wallichii* (syn. *V. jatamansi*) and Sedonium^®^, an ethanol extract of *V. officinalis* [26].

Recently, increasing attention by the scientific community has focused on the EOs of different Valerian species as well as on another rarer species belonging to the same family, Nj. With this purpose, the chemical composition of different species from India and other countries has been investigated [27]. Generally, the yield of the EO from the root/rhizome of these plants, with a typical musky, woody, sweet and balsamic odor, amounts to 0.3–2.1% (*v/w*), although, according to the European Pharmacopoeia, Valerianae radix must contain not less than 0.5% (*v/w*) of EO [20].

The EOs of the species considered have been reported to contain several monoterpenoids and sesquiterpenoids, to which their pharmacological effects, in particular at the CNS level, seems mainly attributable to [7,28,29]. Indeed, some of these compounds have been shown to have a direct activity on the amygdale district or to inhibit the enzyme-induced breakdown of GABA in the brain, resulting in sedation [30]. However, the mechanisms that underline the neuroactive effects of Valerian EOs are yet poorly investigated.

In the past two decades, studies on EOs have focused not only on their pharmacological properties, but also on problems related to quality control and standardization of valerian preparations [31]. In addition, EOs composition and variation are significantly influenced by the growth conditions of the plants (environment, pedo-climatic characteristics and harvesting season) and, of course, by their genetic features [7], as shown by several studies concerning Valerian species [22,32].

Considering this and keeping in view the growing interest on these species, this study was aimed to systematically investigate the botanical and genetic features of these plants by micromorphological and DNA barcoding analyses and subsequently, to characterize and compare the chemical composition and neuroactive effects of EOs isolated by steam distillation from root/rhizome of Vo, Vj and Nj.

## 2. Results

### 2.1. DNA Barcoding Studies

Good DNA quality (i.e., A260/A230 and A260/A280 absorbance ratios within the range 1.8–2.2) and extraction yield (i.e., 20–40 ng/μL) were obtained from Vo and Vj rhizome/roots. Due to the state of conservation of the sample (i.e., very friable rhizome/roots), Nj was treated with a different extraction protocol (CTAB method as described in the Material and Methods Section). Finally, good DNA quality (i.e., A260/A230 and A260/A280 absorbance ratios within the range 1.8–2.2) and extraction yield (10 ng/μL) was obtained also for Nj. Each barcode sequence was taxonomically assigned by using BLASTn analysis to the plant species with the nearest matches (maximum identity >99% and query coverage of 100%). All the samples returned 100% maximum identity (with 100% query coverage). Results are shown in Table 1.

### 2.2. Macro- and Micro-Morphological Studies

**Figure 1:**
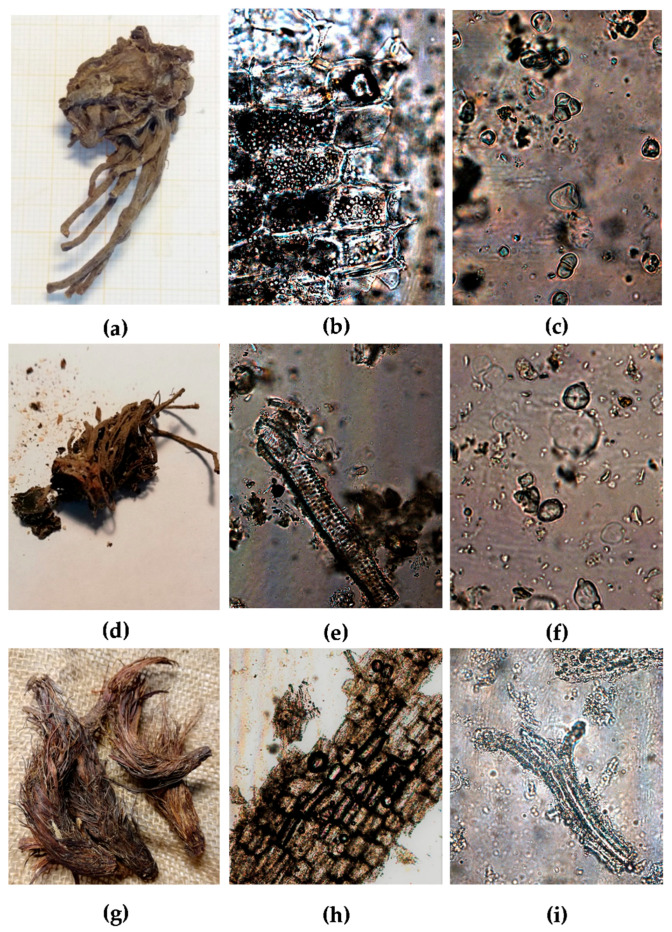
(**a**–**c**) Vo: (**a**) dried root/rhizome; (**b**–**c**) powdered material: (**b**) parenchymatous cells with starch grains (20×); (**c**) starch grains (40×); (**d**–**f**) Vj: (**d**) dried root/rhizome; (**e**–**f**) powdered material: (**e**) scalariform vessels (20×); (**f**) starch grains (40×); (**g**–**i**) Nj: (**g**) dried root/rhizome; (**h**–**i**) powdered material: (**h**) cork cells (10×); (**i**) fibers and small starch grains (40×).

**Figure 2:**
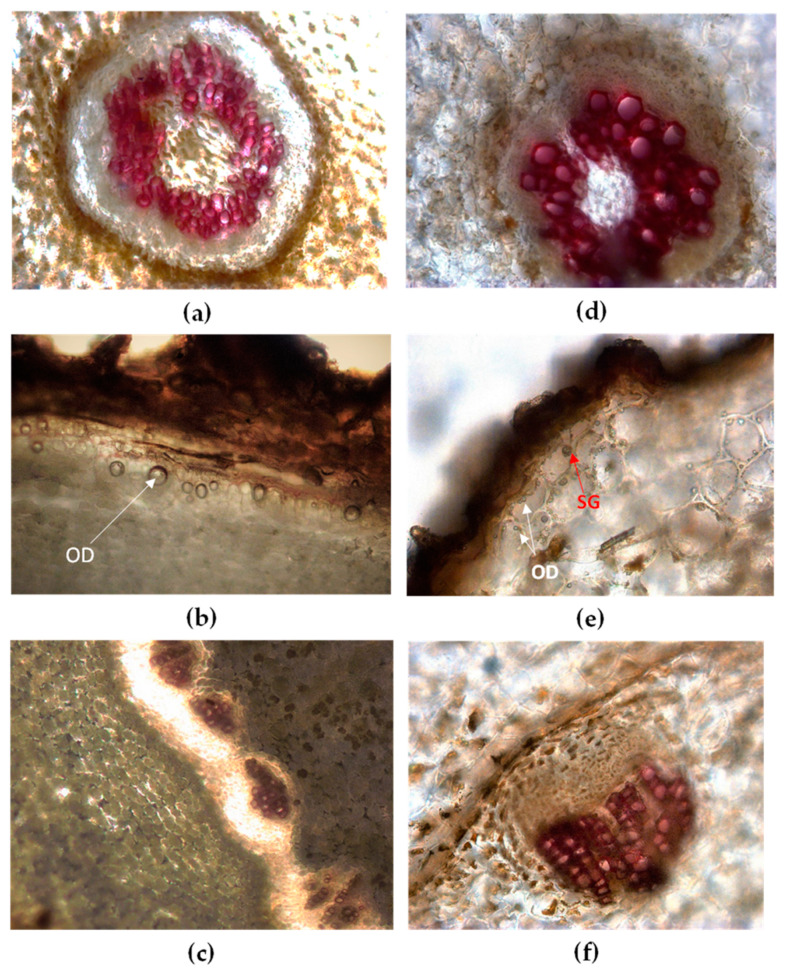
Light microscopy view of root/rhizome transversal sections (TS) of Vo (**a**–**c**) and Vj (**d**–**f**) stained with phloroglucinol-HCl. Vo: (**a**) cortex and stele in older roots; (**b**) under the rhizome cork the outermost layers of parenchymatous cortex contain oil droplets (OD, arrow); (**c**) collateral vascular bundles circularly arranged in the rhizome. Vj: (**d**) cortex and stele in older roots; (**e**) starch grains (SG, arrow) and oil droplets (OD, arrows) are visible within parenchymatous cortex; (**f**) magnification of a singular collateral vascular bundle in the rhizome.

**Figure 3:**
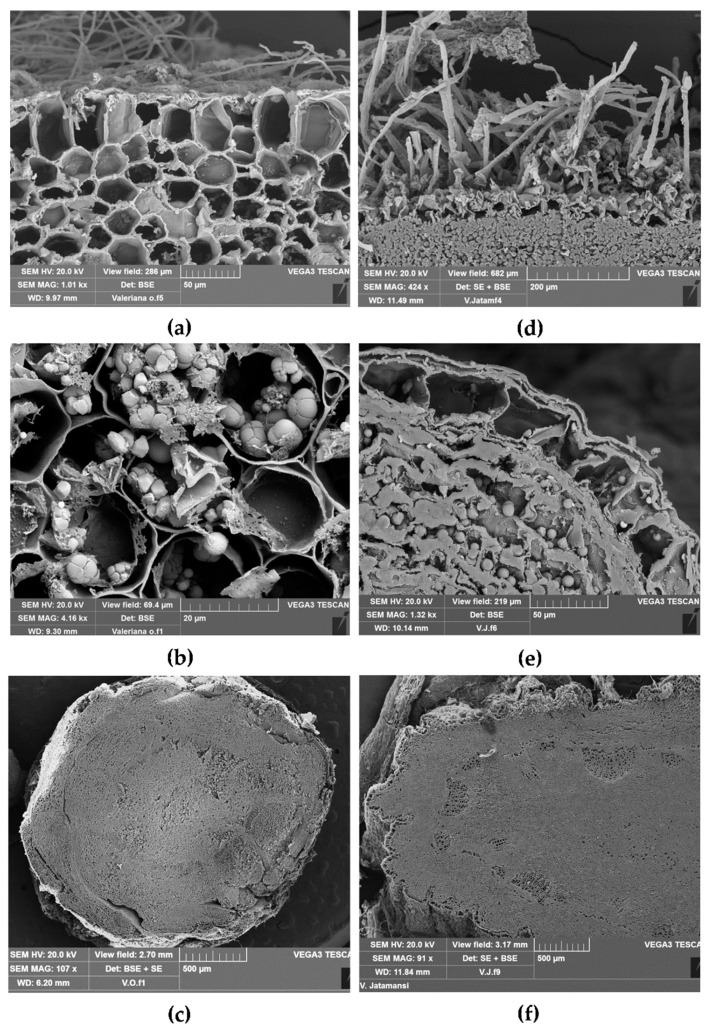
Scanning electron microscopic view of TS of root/rhizome from Vo (**a**–**c**) and Vj (**d**–**f**): (**a**) epidermis with root hairs and the hypodermal layer of the cortex; (**b**) starch occurring as single or compound grains (2–6 components) within cortical parenchymatous cells; (**c**) TS of rhizome showing vascular bundles circularly arranged; (**d**) epidermis with many root hairs and parenchymatous cells filled with starch grains; (**e**) exoderm and parenchymatous cells filled with starch, generally occurring as single or compound grains with two components; (**f**) TS of rhizome with many vascular bundles surrounding the central pith.

**Figure 4:**
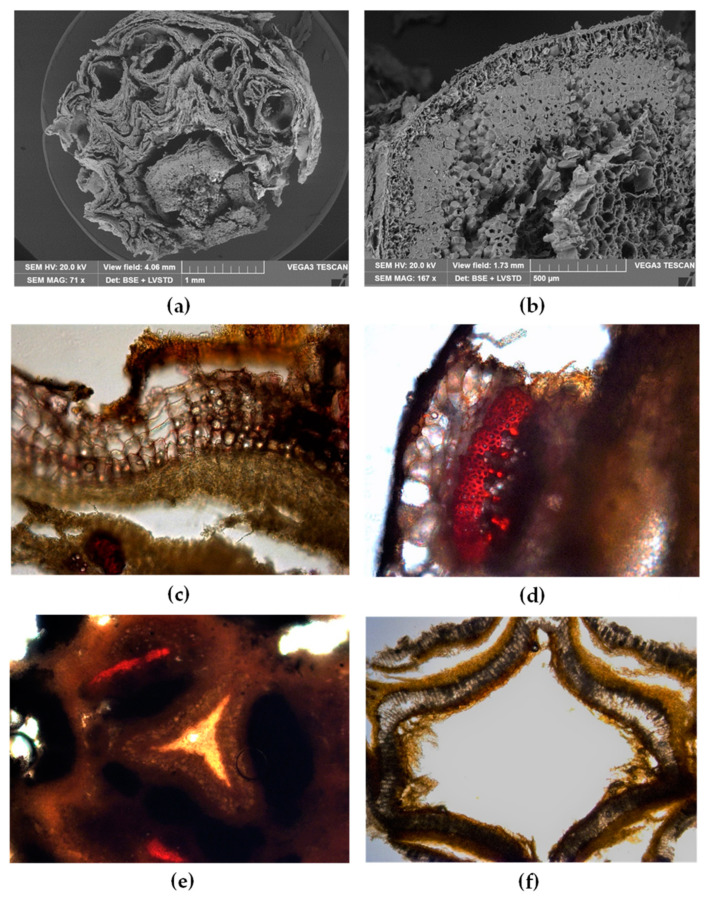
Nj: (**a**,**b**) SEM micrographs, (**c**–**f**) light microscopy observations of TS rhizome stained with phloroglucinol-HCl. (**a**) The rhizome is surrounded by many remains of the basal leaves petioles; (**b**) particular of TS rhizome in which a multi-layered cork and large bundles of sclerenchymatous fibers are visible; (**c**) within suberized cork cells many oil droplets are present; (**d**) bundles of sclerenchymatous fibers appear red-stained by phloroglucinol-HCl; (**e**) the parenchymatous pith showing a characteristic subtriangular-stellate shape is enclosed by cork rings; (**f**) older portion of the rhizome shows a necrotic pith, the cavity of which is surrounded by medullary cork layers.

Most representative features of root/rhizome and powder drugs from the Caprifoliaceae species studied are shown in Figure 1, Figure 2, Figure 3 and Figure 4.

The rhizome of Vo (about 2–5 × 2–3 cm) is conical, dull brown in color, and shows many long stout roots attached (Figure 1a). It has a slightly bitter and spicy taste and a penetrating odor, resembling that of valeric acid and camphor [33].

The rhizome of Vj (about 2.5–12 × 2–3.5 cm) is brownish-black and shows many brown roots (Figure 1d). It has a bitter and slightly camphoraceous taste and a strongly aromatic odor, reminiscent of isovaleric acid [15,33].

The rhizome of Nj (about 2.5–10 × 1–1.5 cm) is elongated, brownish in color, and it appears completely covered with silky reddish fibers that are the remains of the leaf bases (Figure 1g). Its taste is acrid, and the odor is strongly aromatic [15].

Under the microscope, in the pulverized root/rhizome of Vo, Vj, and Nj a few differences can be observed. The powder of Vo is light brown and is characterized by numerous fragments of parenchyma with rounded or elongated cells containing a large number of starch grains (Figure 1b,c). There are also cells containing light-brown resin, rectangular sclereids with pitted walls, xylem isolated or in non-compact bundles, root hairs, and cork fragments [33].

The powder of Vj is dull brownish and shows cork cells in surface view, medullary rays, fragments of fibers, scalariform vessels (Figure 1e), starch grains (Figure 1f), and cells filled with brownish contents [32].

The powder of Nj is brown and shows root hairs, medullary rays, cells filled with reddish-brown contents, cork cells in surface view (Figure 1h), fibers and small starch grains (Figure 1i), xylem vessels with pitted and scalariform secondary wall thickenings [14].

In Figure 2a–f and Figure 3a–f some characteristics of Vo and Vj root/rhizome in transversal sections (TS) are shown.

Vo root has a small central stele surrounded by a monolayer endodermis with suberized and tangentially elongated cells. Phloem and xylem form a continuous ring around the pith (Figure 2a). The cortex is wide and composed of many layers of spheroidal parenchymatous cells filled with a large amount of starch. Starch occurs simple or compound. The hypodermal layer presents large polygonal cells, containing oil droplets (Figure 2b). In TS of rhizome numerous vascular bundles, circularly arranged are visible (Figure 2c and Figure 3c). The rhizome pith is wide and composed of large parenchyma cells, occasionally showing sclereids. The endodermis appears dark brown, surrounded by a broad cortex consisting of spheroidal parenchyma cells with a large amount of starch. A poorly-developed periderm can partly replace the outer layers of rhizome [33,34,35].

Vj root has a circular central stele (Figure 2d) with a small parenchymatous pith, surrounded by a large cortex consisting of many layers of cells rich in starch grains. Remains of the epidermis with root hair are visible and under the dark brown cork lies a hypodermis rich in oil droplets and starch grains (Figure 2e). TS of the rhizome shows many collateral vascular bundles arranged in a circular ring (Figure 2f and Figure 3f). The pith is broad parenchymatous with starch grains, and the endodermis is monolayered (Figure 3e). The cortex is parenchymatous with starch grains oil droplets and a yellowish-brown substance. Oil droplets can be occasionally found also within the suberized cells of the cork [33,34,35].

By scanning electron microscopy (SEM) analysis (Figure 3a–f) some anatomical details of Vo and Vj root/rhizome were clearly highlighted.

In TS of Vo, root epidermis shows many root hairs and small suberized cells; the hypodermis is made up of polygonal-quadratic cells (Figure 3a). According to Houghton 2017 [33], we observed starch occurring as simple or compound grains (with 2–6 components) (Figure 3b). The rhizome of Vo shows a regular outline and internally a circular ring of vascular bundles surrounds the central pith (Figure 3c).

TS of Vj root shows an epidermis with a large number of root hairs and the cells of the parenchymatous cortex filled with starch (Figure 3d). At higher magnification starch generally occurs as single grains, but also compound grains of two components can be found (Figure 3e). Vj rhizome appears lobed in outline in TS and shows many vascular bundles circularly arranged around the medullar pith (Figure 3f).

The very friable rhizome of Nj made it difficult to prepare samples for anatomical studies [9,14,15,36]. Most representative features were pointed out combining light microscopy and scanning electron microscopy (Figure 4a–f).

TS of the dried rhizome is circular in outline and densely covered with many remains of the petiole of the basal leaves (Figure 4a). A multi-layered cork is present (Figure 4b) and light microscopy analysis shows the presence of many oil droplets within the polygonal suberized cells (Figure 4c). Under the cork, many bundles of sclerenchymatous fibers can be observed by both SEM (Figure 4b) and light microscopy (Figure 4d). In light microscopy, these fibers appear red-stained by phloroglucinol-HCl. Inter xylary cork is present and a circular ring of collateral vascular bundles, encircled by cork rings, encloses a large pith star-shaped (Figure 4e). In the older portion, the pith became necrotic and its cavity appears surrounded by medullary cork layers (Figure 4f).

### 2.3. Phytochemical Characterization

The phytochemical profile of the three plant species investigated is reported in Table 2.

As can be seen from the Table 2, which showed the phytochemical profile of the three Caprifoliaceae EOs in comparison, Nj had the most complex chemical profile with 69 identified compounds, followed by Vj and Vo, with 42 and 39 identified compounds, respectively.

Sesquiterpene hydrocarbons were the most abundant compounds identified both in Nj and Vj (61.59% vs. 67.23%, respectively) followed by oxygenated sesquiterpenes (23.93% vs. 30.45%). However, the main difference found in the phytochemical profile of Nj in comparison with Vj was the higher percentage of monoterpenes (4.83% vs. 0.67%), as well as the presence of other compounds such as aldehydes and ketones (8.01% in Nj EO) that were completely lacking in Vj EO.

On the contrary, esters were present in both EOs, although predominantly in Vj EO (0.82% vs. 0.42% in Nj).

Vo showed a completely different phytochemical profile from the other EOs, with oxygenated monoterpenes as the most abundant class (53.29%), followed by monoterpene hydrocarbons (23.70%), and other compounds, in particular esters (14.71%), and an almost superimposable amount of sesquiterpene hydrocarbons and oxygenated sesquiterpenes (4.08% and 4.44%, respectively).

However, beyond the different classes of compounds identified, the three EOs showed substantial differences in the most representative compounds as well as in their relative abundance.

Jatamansone (13.96%), calarene (8.22%), valencene (8.05%), seychellene (6.90%), γ-gurjunene (6.36%) and aromadendrene (4.17%) were the most abundant compounds identified in Nj EO, whereas maaliol (17.43%), γ-gurjunene (11.88%), calarene (10.57%), δ-guaiene (8.53%), aromadendrene (6.43%) and β-patchoulene (6.02%) were the most abundant compounds identified in Vj EO (Table 2). Conversely, bornyl acetate (46.90%), camphene (13.85%), trans-valerenyl acetate (13.18%), mirtenyl acetate (3.94%), α-pinene (3.39%) and β-pinene (2.76%) were the most abundant compounds in Vo EO.

**Figure 5:**
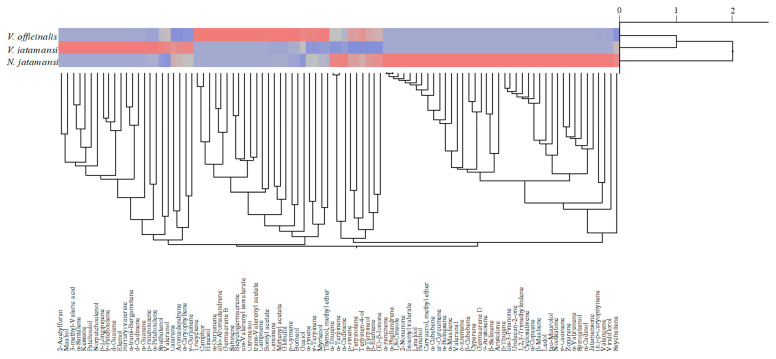
Agglomerative hierarchical clustering analysis of the three Caprifoliceae essential oils (EOs) investigated: Vo, Vj and Nj.

The dendrogram depicted in Figure 5 showed the results of agglomerative hierarchical clustering analyses performed on the 95 compounds identified into the three EOs of the Caprifoliaceae species investigated. Two clusters were identified: one comprising Vo and Vj and another one for Nj. Further analyses, such as two-way clustering, showed how some components were mainly present in one species rather than in another.

Indeed, using the color map it has been possible to identify where a specific constituent was mainly represented. In particular, the blue color was indicative of the complete absence of the component in the corresponding species; the grey color was indicative of a very low presence, whereas the red one was indicative of a relevant presence of this particular compound. The most intense shade of red was indicative of a higher percentage of the compound (Figure 5).

For example, patchoulol was found only in Vj EO, whereas jatamansone and trans-valerenyl acetate were found exclusively into Nj and Vo EOs, respectively.

Therefore, this type of analysis could be a useful tool to quickly identify chemotaxonomic markers useful not only to discriminate between different plant species but also to discover any adulteration such as the well-known between Vj and Nj [15].

### 2.4. Acetylcholinesterase Inhibitory Activity

In the present study, the effects of Nj, Vj and Vo EOs were evaluated against the acetylcholinesterase (AChE) activity. All investigated EOs showed a concentration-response relationship (R^2^ ≥ 0.975, data not shown), within the concentration range tested (17.50–140.0 μg/mL for Vo and Nj, and 35.00–280.0 μg/mL for Vj) with the following half maximal inhibitory concentrations (IC_50_) with respective confident limits (C.L.) calculated at 95%: Nj 67.15 µg/mL (C.L. 95%, 57.47–78.44), Vo 127.30 µg/mL (C.L. 95%, 99.30–163.20) and Vj 246.84 µg/mL (C.L. 95%, 191.86–317.58). Nj showed the strongest AChE-inhibitory activity followed by Vo and Vj (*p* < 0.001), although results were significantly different compared with the positive control galantamine, which showed an AChE-inhibitory activity equal to 97.14% at the highest concentration tested (7 µg/mL). 

### 2.5. Neuroactive Effects

**Figure 6:**
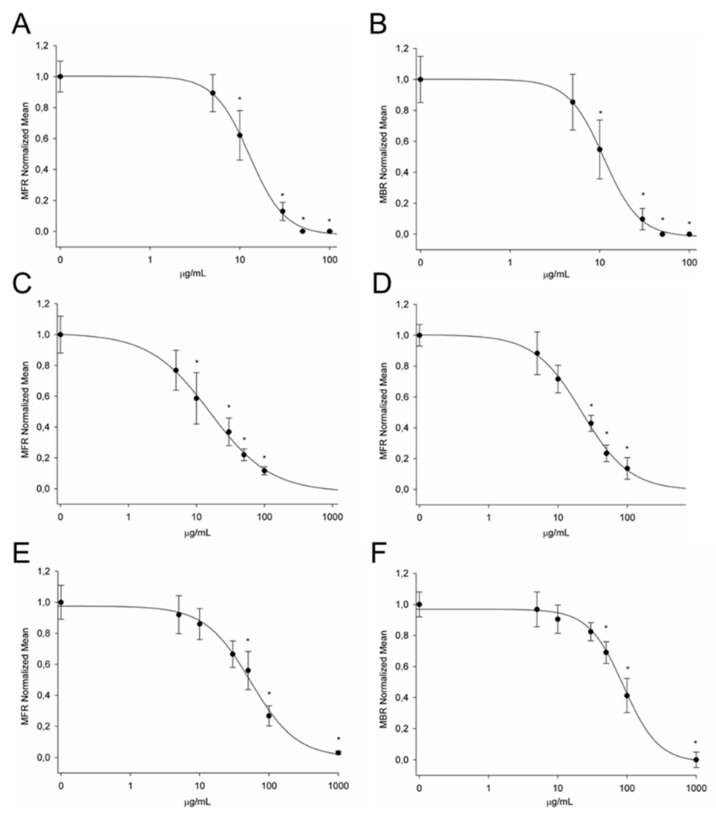
Effects of increasing concentrations of Nj, Vo and Vj EOs on cortical neuron networks activity. Nj EO showed a concentration-dependent inhibition for both mean firing rate (MFR) (**A**) and mean burst rate (MBR) (**B**), with IC_50_ values of 12.8 and 11.1 µg/mL, respectively (n = 6). The EO of Vo showed similar but less strong effects on MFR (**C**) and MBR (**D**), with IC_50_ values of 16.0 and 22.2 µg/mL, respectively (n = 5). Vj EO was the less effective to induce the inhibition of neuronal activity with IC_50_ values of 54.4 and 88.7 µg/mL for MFR (**E**) and MBR (**F**), respectively. * *p* < 0.05.

In this study, changes in the spontaneous electrical activity of in vitro cortical neuronal networks in response to Nj, Vj and Vo EOs were recorded using multielectrode chips. The data analysis described MFR and MBR parameters characterizing the changes in overall neuronal activity state and its bursting behavior. The concentrations used for Nj and Vo EOs were of 5, 10, 30, 50, 100 μg/mL while those for Vj OE were of 5, 10, 30, 50, 100, 1000 μg/mL. All concentrations were cumulatively administrated in the MEA chip to obtain the concentration-response curves for MFR and MBR parameters. For all three EOs, the application resulted in a concentration-dependent decrease in the electrical activity of neuronal networks until the complete loss of activity. As shown in Figure 6, the Nj EO was the most potent in the inhibition of spontaneous activity with an IC_50_ of 12.8 and 11.1 μg/mL for MFR and MBR, respectively. A slightly lower efficacy, without any statistically significant difference from the previous one, was obtained with the administration of Vo EO, which inhibited electrical activity with an IC_50_ of 16.0 and 22.2 μg/mL for MFR and MBR, respectively. Differently, Vj EO induced a decrease of neuronal activity at higher concentrations as observed by the IC_50_ of 54.4 and 88.7 μg/mL for MFR and MBR, respectively. Therefore, comparing the IC_50_ values of the three Caprifoliaceae EOs, only Vj was significantly different from the other two (*p* < 0.01). As a positive control, we assessed the effects of the non-selective GABA agonist muscimol, which has been previously shown to inhibit electrical activity developed by cortical neuronal networks grown on MEA [37]. We obtained an IC_50_ of 0.28 µM for MFR and of 0.32 µM for MBR, in line with that previously determined.

## 3. Discussion

DNA barcoding led to correct identification of the expected species allowing discrimination between Vo, Vj and Nj. For Nj, it has been difficult to obtain DNA, due to the very friable consistency of the dried rhizome. Nevertheless, all the samples were identified successfully. This indicates that DNA barcoding is a powerful tool for species identification, to prevent fraud and substitutions that are common for the roots of Vo and Nj.

According to WHO, the first step of botanical standardization of a medicinal plant consists of macroscopic and microscopic studies. However, dried root and rhizome represent a recalcitrant material for this kind of study, especially when they show a very friable consistency. Nevertheless, the optical microscope observation combined with SEM analysis could be of help to obtain the characterization of these botanicals, allowing the analysis of both sectioned and powdered materials. In the present study, the macro- and micro-morphological elucidation of three Caprifoliaceae species, viz. Vo, Vj, Nj was carried out as the first step to authenticate their identity. Comparing the three rhizomes, the most significant feature was the macro- and microscopic appearance of Nj, which was always densely covered with silky reddish fibrous remains of the petiole of the basal leaves. This herbal drug obtained from a rare Himalayan species is an important ingredient of Ayurvedic medicine, but because of its high price, it is frequently adulterated with other species such as the related Caprifoliaceae species, like *Valeriana* spp., or plants belonging to other families, for instance, *Selinum vaginatum*, belonging to the Apiaceae [15].

With regards to microscopic observations, the rhizome section from Nj also showed a peculiar shape of the medullary pith that appears star-shaped and turns necrotic in the older portions. On the contrary, the rhizomes from Vo and Vj had very similar macroscopic features, showing an intact anatomical structure even in the oldest portions. Another interesting feature was the presence of starch granules, generally occurring as simple or compound granules with few components in Vj and Nj, whereas in Vo compound granules with many components were very frequent.

The phytochemical characterization of the three Caprifoliaceae EOs allowed the identification of 95 compounds, some of which were specific to each EO, while others were identified for the first time (Table 2). The EOs of Vo e Vj are certainly the most thoroughly investigated, whereas Nj EO is still little investigated.

Few studies are currently available about the chemical composition of Nj EO [38,39,40]. Maiwulanjiang et al. [38] identified only nine compounds (Table 3) with 72.2% of the constituents, which remained unidentified. Nevertheless, there were some chemical compounds in common with the Nj EO investigated in the present study, as shown in Table 3. However, nothing is known about the origin of the plant that was purchased from Hong Kong’s herbal market [38]. This is a fundamental aspect in order to make an appropriate comparison of the phytochemical profile of the EOs, since it is well known that the EO yield and composition change according to ecotype, climatic conditions and environmental interactions.

On the contrary, Costa and co-workers [39] identified, by GC-MS analysis, 40 compounds in an Nj EO coming from Nepal, with a very similar phytochemical profile in comparison with the EO investigated in the present study (Table 3). According to our results, patchoulol was absent, confirming that the origin of the plant certainly influences the expression of secondary metabolites and in particular of the most abundant compounds.

Indeed, Chauhan and co-workers [40], who investigated the variations in the EO composition of Nj in samples collected from two different locations (North and South) of Tungnath in Rudraprayag district of Uttarakhand (India), found a different phytochemical profile. They found 41 compounds, of which 19 in common with the Nj EO here investigated (Table 3), but with patchoulol as the most abundant compound (40–52%) [40].

Regarding Vj, several studies about the phytochemical characterization of EOs distilled by plants coming from different locations were carried out [7,20,27,32]. Verma and co-workers [27] analyzed the EO composition of 17 Indian Vj populations collected from different locations of Uttarakhand (Wester Himalaya). They identified 39 compounds, of which 22 were superimposable with our EO (Table 3). However, the most representative compounds in Indian VJ EO were completely different from those identified in the present study, with patchoulol (18.1–66.70%), α-bulnesene (*tr*-23.5%), α-guaiene (0.2–13.3%), seychellene (0.2–9.9%), viridiflorol (*tr*-7.3%) and δ-cadinene (*tr*-3.5%) as the most abundant compounds.

However, also Bhatt and co-workers [7] investigated the chemical composition of the EO of an Indian Vj collected from Katarmal forest (Almora). The authors identified 20 compounds and among these, 15 were found also in the Vj EO here investigated (Table 3) with δ-guaiene (10.04%), seychellene (4.83%), α-guaiene (3.86%), α-humulene (3.96%) and α-patchoulene (2.38%), that were found in comparable amounts.

Raina et al. [20] investigated the EO composition of several Vj accessions collected from five districts of Uttarakhand (North-East India) situated at different geographical locations and one accession collected from Shillong district of Meghalaya (North-East India).

They identified 21 compounds in the six different locations selected, although with very high variability in terms of metabolites expression. In general, the phytochemical profile identified is quite similar to that found in our sample (Table 3).

Sing and co-workers [32] analyzed the chemical profile of Vj EOs coming from different location/regions of Himachal Pradesh (India). They identified 23 compounds. Among them, 12 compounds were identified also in our Vj EO (Table 3). Among the most abundant compounds identified, calarene (3.16–9.18%), α-patchoulene (0.74–4.98%) and seychellene (0.86–1.43%) were found at highest percentage in our Vj EO (10.57%, 3.35% and 4.27%, respectively) [32]. On the contrary, viridiflorol and patchoulol were much less expressed (0.16% and 0.41%, respectively) [32].

Considering this, beyond the high variability found in the phytochemical profile of plants coming also from different locations/regions of the same country, what emerges overwhelmingly is that Indian Vj is characterized by high amounts of patchoulol, whereas this metabolite is poorly expressed in our sample coming from Nepal, according to previous studies [39].

Comparative analyses between EOs of Vo plants coming from different countries were carried out. Safaralie and co-workers [41] identified 53 compounds into Iranian Vo EO, of which 25 were also found in our Vo EO (Table 3). According to our results, bornyl acetate (11.6%) is the most abundant compound identified in hydrodistilled EO, followed by acetoxyvaleranone (7.6%), β-caryophyllene (5.1%), spathulenol (4.7%), valeranol (4.3%) and *allo*-aromadendrene (4.1%). Among these, acetoxyvaleranone and valeranol were absent in our Vo EO, but bornyl acetate (46.90%), as well as the class of monoterpenes (76.99% vs. 5.1%), were much more expressed. The main components of Iranian Vo EO were investigated also by Samaneh et al. [31]. They identified 21 compounds, of which 11 were in common with our Vo EO (Table 3). However, despite the same provenance, the phytochemical profile is completely different from that elucidated by Safaralie et al. [41].

Conversely, Wang et al. [42], which analyzed the chemical composition of a Vo EO coming from China found, according to our results, a higher content of monoterpenes (37.26%). Authors identified only 17 compounds, of which seven were in common with the previous one (Table 3). However, the most abundant compounds completely differ from those reported in the present and previous studies, because they identified as the most representative compounds patchoulol (16.75%), α-pinene (14.81%), β-humulene (8.19%), α-bulnesene (7.10), bornyl acetate (6.73%), limonene (6.56%) and camphene (6.51%).

Among other things, patchoulol as predominant compound is a very strange result, because this metabolite has not been identified neither in the present study nor in the previous ones concerning Vo EOs.

The cholinergic disturbance is often at the base of neuropsychological impairments typical of several neurological disorders such as Alzheimer’s disease.

Several aromatic plants have been used worldwide to alleviate and cure neuronal ailments and EOs and their-isolated bioactive compounds could represent new therapeutic approaches [43,44].

Several extracts, as well as some EOs and isolated compounds (mainly monoterpenes and sesquiterpenes) from *Valeriana* and *Nardostachys* genus, have shown AChE inhibitory activity [42,45,46,47,48,49].

Some Indian medicinal plants were screened for AChE inhibitory activity by Mukherjee et al. [45]. Authors found that rhizome hydroalcoholic extract of Nj showed a strong AChE-inhibitory activity (IC_50_ 130.11 µg/mL). According to this result, another in vitro screening of 20 methanol extracts of ayurvedic medicinal plants used for cognitive disorders highlighted that Nj was among the most powerful AChE inhibitors (IC_50_ 40.5 vs. 0.075 µg/mL of the positive control physostigmine) [47] with IC_50_ value in the same order of that found in the present study (IC_50_ 67.15 µg/mL).

Several neurological effects of Nj are known such as GABA enhancing effects and its useful role in preventing cerebral ischemia [50]. Moreover, jatamansone, the most abundant compound of its EO, has shown tranquilizing effects on mice and monkeys and it was also found to impair biosynthesis of serotonin in the rabbit brain, decreasing 5-hydroxytryptamine level [51].

Considering this, jatamansone could play a pivotal role also in the AChE inhibitory activity of the Nj EO and further studies with the purified compound are needed to support this hypothesis.

Previous studies reported that the chloroform and ethyl acetate fractions of Vj exhibited significant AChE-inhibitory effects (IC_50_ 59 and 61 µg/mL, respectively) [52]. However, several bioactive compounds such as some iridoids and sesquiterpenoids, isolated from ethyl acetate fraction, displayed no activity suggesting a synergistic effect [48]. On the contrary, its EO displayed only a weak inhibitory activity [53]. These results are in accordance with ours, where Vj EO showed the lowest AChE-inhibitory activity (IC_50_ 246.84 µg/mL) in comparison with Nj (IC_50_ 67.15 µg/mL) and Vo EOs (IC_50_ 127.30 µg/mL).

Several well-known compounds as well as two new guaiane-type sesquiterpenoids (valerol A and kessyl 3-acetate) isolated from the roots of Vo were tested for inhibitory activity on AChE [46]. Only four compounds showed AChE-inhibitory activity at 100 µM. Among these, spatulenol, identified also in the Vo EO here investigated, showed the strongest inhibitory activity with an inhibition percentage (I%) of 49.1% [46].

This is the first study in which the AChE-inhibitory activity of Vo, Vj and Nj were evaluated and compared. Studies concerning both EOs and their most abundant pure compounds (monoterpenes and sesquiterpenes) are rather scarce. Nevertheless, it is possible to postulate that isolated compounds, mostly oxygenated and belonging to the class of sesquiterpenes, showed a weak inhibitory activity on AChE, while it is the plant complex, extract or EO, which exhibits the greatest inhibitory activity on this enzyme. This is probably attributable to a synergistic action not only of the most representative compounds of the plant complex but also to the presence of minor compounds, often specific to each species taken into consideration.

Previous functional screening of traditional herbal antidepressants was performed with primary cortical neuronal networks grown on multielectrode neurochips. Authors reported that the multiparametric assessment of electrical activity changes caused by a mixture containing Vo and other psychoactive herbal extracts revealed a receptor-specific and concentration-dependent inhibition of the firing patterns. Moreover, they showed evidence that the herbal extracts acted on GABA and serotonin (5-HT) receptors, which are recognized targets of pharmacological antidepressant treatment [54]. These results are in line with those described in this study in which a concentration-dependent inhibition of spontaneous electrical activity for Vo and Nj EOs, was obtained. In particular, similar concentration-response curves for MFR and MBR was observed suggesting that they have a similar mode of action.

However, the dissimilar chemical profile of Nj and Vo did not allow to speculate about any compound potentially responsible for the significant effect on electrical activity induced by the two EOs. For this purpose, further experiments will have to be carried out using the most abundant pure compounds present in the EOs in order to detect which is responsible for inhibiting the neuronal activity.

On the contrary, the Vj EO was the least effective in inhibiting the spontaneous electrical activity on our neuronal cultures and the two derived activity parameters were affected with different potency. This strongly suggests that Vj EO acts through different targets that affect the number of spikes in a burst.

## 4. Materials and Methods

### 4.1. Chemicals

C7-C40 saturated alkane standard, acetylthiocholine iodide (ATCI), 5,5′-dithiobis (2-nitrobenzoic acid) (DTNB), AChE, galanthamine, sodium phosphate dibasic (Na_2_HPO_4_), potassium phosphate monobasic (KH_2_PO_4_), sodium chloride (NaCl) and potassium chloride (KCl) were purchased from Sigma-Aldrich (Milan, Italy). α-Pinene, camphene, β-pinene, limonene, 1-8-cineole, linalool, borneol, terpinen-4-ol, citronellol, bornyl acetate, β-caryophyllene, aromadendrene, α-humulene and guaiol were purchased from Extrasynthese (Genay, France). Dichloromethane was GC-grade and was purchased from Merck (Darmstadt, Germany). All other chemicals and solvents were of analytical grade.

### 4.2. Plant Material and Essential Oils Preparation

Rhizomes/roots of spontaneous plants of Vj and Nj were collected in October 2019 in Darchula District, Sudurpashchim Pradesh, Nepal (29°50′54.47″ N, 80°32′36.21″ E). The identity of the collected specimens was confirmed by Dr. Dipesh Pyakurel, a botanist from Nepal, through specialized literature [55,56]. Voucher specimens were deposited at Tribhuvan University Central Herbarium (TUCH) of Nepal with the following voucher numbers: DA 17 for Nj and DA 29 for Vj. Plant nomenclature follows Plant of the World [57].

Rhizome/root from Vj and Nj were air-dried, chopped (100 g) and steam-distilled in loco by G.G. of Nepal Herbs and Herbal Products Association (NEHHPA), until no significant increase in the EO volume was observed (4 h). The EOs were dehydrated on Na_2_SO_4_ and stored in burnished vials with nitrogen headspace at 4 °C until analysis. The EO yields, calculated on the fresh-weight basis (% *v*/*w*) were 1–1.5% and 1–1.2% for Vj and Nj, respectively.

Micromorphological characterization and DNA barcoding analyses of roots/rhizomes of Vj and Nj were carried out on samples of the same plants, which were provided by G.G. of NEHHPA.

Commercial samples of Vo root/rhizome and of its EO, derived from plants of Chinese origin, were kindly provided by Bios Line S.p.A. (Padova, Italy), and were used for micromorphology and DNA barcoding as well as for EO phytochemical characterization.

### 4.3. Species Identification by DNA Barcoding

Fifty milligrams of dried plants (i.e., Vo, Vj and Nj) were treated for DNA extraction by using the DNeasy Plant Mini Kit (QIAGEN, Hilden, Germany). One gram of dried plant (i.e., Nj) was treated for DNA extraction by using the CTAB method [58]. DNA for each sample was checked for concentration by using a Qubit 2.0 Fluorometer and Qubit dsDNA HS Assay Kit (Invitrogen, Carlsbad, CA, USA).

The chosen DNA barcoding region was the intergenic spacer *psbA-trnH* thanks to its high power of discrimination. Primer details are described in Frigerio and colleagues [59].

PCR amplification was carried out using puReTaq Ready-To-Go PCR beads (GE Healthcare Life Sciences, Italy) in a 25 μL reaction according to the manufacturer’s instructions. PCR cycles consisted of an initial denaturation step for 7 min at 94 °C, followed by 35 cycles of denaturation (45 s at 94 °C), annealing (30 s at 53 °C) and extension (1 min at 72 °C), and a final extension at 72 °C for 7 min as described in Frigerio and colleagues [60]. Amplicons presence was assessed by electrophoresis on agarose gel (i.e., 1.5%). Purified amplification products were bidirectionally sequenced using an ABI 3730XL automated sequencing machine at Eurofins Genomics (Ebersberg, Germany). The 3’ and 5’ terminal portions of each sequence were clipped to generate consensus sequences for each sample. All sequences were manually edited, the primer was removed and after pairwise alignment, the obtained sequences were identified by a standard comparison approach against a GenBank database with Basic Local Alignment Search Tool (BLAST) (https://blast.ncbi.nlm.nih.gov/). Each barcode sequence was taxonomically assigned to the plant species with the nearest matches (maximum identity >99% and query coverage of 100%) according to Bruni and colleagues [61].

### 4.4. Micromorphological Characterization

For light microscopical studies of plant material, the standard methods for authentication of botanicals were used. Specimens of about 3–4 cm of root/rhizome from the three species were soaked in hot water for 10 min, then hand sectioned using a razor blade. Furthermore, pulverized materials were obtained using a mortar and pestle. Subsequently, both sections and powders were: (a) mounted in water and directly observed; (b) clarified with chloral hydrate solution for 15 min, rinsed in water and observed; (c) stained with phloroglucinol and HCl for showing lignin.

For scanning electron microscopy (SEM) analysis, samples of about 2 cm long were fixed overnight at 4 °C in FineFIX working solution, prepared with 70% ethanol dilution of the concentrated product (Milestone s.r.l., Bergamo, Italy), according to Chieco et al. [62]. Specimens were then dehydrated through a graded series concentration of ethanol (75, 85, 95, 100%) and finally in CO_2_ by a Critical Point Drier apparatus (K850 CPD 2M Strumenti S.r.l., Roma, Italy). Dried samples were hand sectioned, mounted on stubs and coated with 10 nm gold. The observation was carried out with aVega3 Tescan LMU SEM (Tescan USA Inc., Cranberry Twp, PA, USA) at an accelerating voltage of 20 kV.

### 4.5. Phytochemical Analyses

The chemical characterization was carried out by an Agilent gas chromatograph (GC, 7890A), equipped with a flame ionization detector (FID) (Agilent Technologies Santa Clara, CA, USA). The separation was carried out using an HP-5MS capillary column (30 mm, 0.25 mm coated with 5% phenyl methyl silicone, 95% dimethyl polysiloxane, 0.25 μm film thickness) and helium as carrier gas (1 mL/min). One microliter of 10% EO/CH_2_Cl_2_ *v/v* solution was injected in split mode (100:1) setting the injector and detector temperature at 250 and 280 °C, respectively. Elution was carried out according to the following program: 60 °C for 6 min, increased to 270 °C at 3 °C/min and held at 270 °C for 4 min [63].

Gas chromatography-mass spectrometry (GC-MS) analysis was carried out on the same instrument, coupled with a mass detector (5975C), by using the same column and operative conditions reported above for analytical GC, but setting the ionization voltage, the electron multiplier and the ion source temperature at 70 eV, 900 V and 230 °C, respectively.

EO constituents were identified by comparison of the GC retention index with those of C7-C40 n-alkanes, with literature data [64], matching the mass spectral data with MS library NIST 08 [65], by comparison of MS fragmentation patterns with those reported in the literature, and by co-injection with commercially available terpene standards.

Quantification was carried out by extrapolation of the compound’s peak areas from GC-FID profiles.

### 4.6. Acetylcholinesterase Inhibition Assay

The AChE inhibitory activity was evaluated according to Smeriglio et al. [43] by using a blank control, a blank, a test, and a test control, each in triplicate. Three independent experiments were carried out. Briefly, in the test group, 400 μL of phosphate-buffered saline (PBS, pH 8.0), 100 μL of EO diluted in methanol (final concentration 17.50-140.0 μg/mL for Vo and Nj, and 35.00-280.0 μg/mL for Vj), 100 μL of 0.4 U/mL AChE and 200 μL of DTNB (0.6 mmol/L) were incubated for 10 min at 37 °C. After that, 200 μL of ATCI (0.6 mmol/L) was added to the reaction mixture and incubated for 30 min. Finally, 500 μL of anhydrous EtOH was added to stop the reaction and absorbance was recorded at 412 nm by a UV-Vis Spectrophotometer (Shimadzu UV-1601).

In the test control group, 100 μL of PBS was used instead of AChE solution. In the blank group, MeOH was used instead of the sample. In the blank control group, 100 μL of MeOH and 100 μL of PBS were used instead of sample and AChE solution, respectively. Galantamine was used as reference compound with well-known AChE inhibitory activity (final concentration 0.88-7.0 μg/mL).

Results were expressed as inhibition percentages, calculated according to the following equation (Equation (1)):

Inhibitory activity (%) = [(Abg − Abcg) − (Atg − Atcg)]/[Abg − Abcg] × 100(1)

where Abg, Abcg, Atg and Atcg are the absorbance of the blank group, blank control group, test group and test control group, respectively. IC_50_ (µg/mL) with C.L. at 95% were calculated by Litchfield and Wilcoxon test, using PHARM/PCS software version 4 (MCS Consulting, Wynnewood, PA, USA).

### 4.7. Microelectrode Array Analysis

#### 4.7.1. Primary Mouse Cortical Neuronal Cultures

All animal procedures were performed according to the guidelines of the Italian Ministry of Health (D.L. 26/2014) and the European Parliament directive 2010/63/EU and complied with all required animal use guidelines. Briefly, the cerebral cortices were isolated from C57BL/6 embryonic day-15 mice, (OF) either sex, and mechanically dissociated in 5 mL of Hank’s Balanced Salt Solution without Ca^2+^ and Mg^2+^ (Thermo Fisher Scientific, Waltham, MA, USA) and the cell homogenate would be allowed to settle out. Then, the supernatant was discarded and the pellet gently resuspended in fresh neurobasal medium (NB; Thermo Fisher Scientific, Waltham, MA, USA) supplemented with 2% B27 (Thermo Fisher Scientific, Waltham, MA, USA) and 1% glutamine (Sigma Aldrich, Milan, Italy). After opportune dilution, 30 μL of cell suspension containing 50,000–60,000 cells were left to settle in the center of each MEA chip. After allowing 1 h for cells to attach, 1000 µL of pre-warmed fresh culture medium was added to each well.

#### 4.7.2. Data Recordings, Signal Processing and Data Analysis

Glass 60-electrode MEA chips have been employed with 30 μm diameter electrodes arranged in an 8 × 8 square grid excluding corners, and 200 μm inter-electrode spacing with an integrated internal reference electrode (60MEA200/30iR-Ti-gr; Multichannel Systems GmbH, Reutlingen, Germany). Before plating the cells, the MEA chip was coated with 0.1% polyethylenimine (PEI P3143, Sigma Aldrich, Milan, Italy) dissolved in borate buffer. The spontaneous neuronal activity was recorded by the USB MEA 120 INV 2 BC System (MCS GmbH, Reutlingen, Germany). The MEA chips were placed into the MEA Amplifier (Gain 1000×) and data were recorded by the MC_Rack software (MCS GmbH, Version 4.4.1.0) at a sampling rate of 10 kHz. A bandpass digital filter (60–4000 Hz) was applied to the raw signal to remove electrical background noise. The spike detection threshold applied was 5.5 times the standard deviation of the mean square root noise. The system also includes a heating system connected to a temperature controller (TC02, MCS GmbH) that keeps the MEA chamber at 37 °C.

Neuronal cultures were recorded from the 3rd to the 5th week in vitro. The experiments were performed on different days using cultures from a minimum of two different isolations. At the beginning of the experimental session, 20 min recording of spontaneous activity in the absence of any compound (control condition) followed by 20 min experimental episodes for each increasing concentration was performed. The experimental protocol is a “cumulative treatment”, and it consists of the administration of 5–8 serial concentrations of each mixture.

To describe the neuronal network activity, we considered these two parameters: the mean firing rate (MFR; number of spikes/s) and the mean burst rate (MBR; number of bursts/min). The analysis was conducted with NeuroExplorer software (Blackrock Microsystems, Salt Lake city, UT, USA) in which the following burst definition parameters were set: bin size = 1 s; maximum interval of starting a burst = 0.01 s; maximum interval of ending a burst = 0.075 s; minimum burst interval = 0.1 s; minimum burst duration = 0.02 s; minimum of number of spikes in burst = 4. Only electrodes with >2 bursts/min were included in the analysis.

Stock solutions of the EOs were prepared in dimethyl sulfoxide (DMSO) with a final concentration of 100 mg⁄mL. During the experiment, DMSO concentrations in the medium did not exceed 0.4%; up to 1% DMSO was shown to not affect the neuronal activity (data not shown).

Data were expressed as the mean and S.E.M. of at least 5 independent experiments, concerning the normalized baseline values obtained in control condition before exposure.

The estimated IC_50_ values were obtained interpolating the normalized concentration-response curves with the following four-parameter logistic function (Equation (2)):

f(x) = Max + (Min-Max)/(1 + (ε/x) β)(2)

where the variable x is the concentration of the compound; the parameter Min is the minimum effect; the parameter Max is the maximum effect; the parameter ɛ is the concentration at the inflection point of the concentration-response curve, i.e., the concentration at which the effect is reduced by 50% (IC_50_); β is a parameter related to the maximum slope of the curve, which occurs at concentration ɛ.

### 4.8. Statistical Analysis

Results were analyzed by one-way analysis of variance (ANOVA). The significance of the difference from the respective controls for each experimental test condition was assayed by using Tukey’s test for in vitro cell-free based assay and phytochemical analyses, and the Holm–Sidak test for cell-based assays using the SigmaPlot 12.0 software (Systat Software Inc. San Jose, CA, USA). Statistical significance was considered at *p* < 0.05.

Moreover, agglomerative hierarchical clustering analysis (AHC) was carried out to highlight the phytochemical relationships and similarities among the different plant species investigated using the statistical JMP7 for SAS software (version 7, SAS Institute Inc., Cary, NC, USA). Euclidean distances were used to measure the dissimilarity between samples, whereas hierarchical clustering was performed according to Ward’s minimum variance method.

## 5. Conclusions

In conclusion, the present study shows that microscopic analyses combined with DNA barcoding represent a rapid and valid approach for herbal drug identification, allowing discrimination of genera and species. Moreover, these techniques, combined with the phytochemical fingerprinting obtained from the EOs are important tools to avoid the adulteration of these herbal drugs and to discriminate between EOs of plants coming from different sites. It is well-known, indeed, that EO yield and composition change according to ecotype, climatic conditions and environmental interactions and that these events strongly influence the biological effects observed.

Finally, this is a very innovative study because it demonstrates experimentally, for the first time, the effects on the central nervous system of *V. officinalis*, *V. jatamansi* and *N. jatamansi* EOs by both AChE inhibitory activity and a reconstituted murine neuronal network in vitro. This reliable and reproducible model reduces the number of animals used to a minimum, according to the principles of the 3Rs (Replacement, Reduction and Refinement) and represents an absolute innovation in the pharmacological/toxicological field to investigate the effects of plant complexes on the central nervous system. Therefore, our methods can be recommended for the correct identification of herbal drugs and the evaluation of the effectiveness of EOs used in therapeutic treatment of nervous disorders.

## Figures and Tables

**Table 1:** DNA barcoding results. In the table are shown declared species, resulted species, origin, collection year and accession numbers deposited in the EMBL Nucleotide Sequence Database (www.ebi.ac.uk/embl).

Declared Species	Resulted Species	Origin	Collection Year	Accession Number
*V. officinalis*	*V. officinalis*	China	2019	LR861814
*V. jatamansi*	*V. jatamansi*	Darchula District, Nepal	2019	LR861815
*N. jatamansi*	*N. jatamansi*	Darchula District, Nepal	2019	LR861816

**Table 2:** Chemical composition of Vo, Vj and Nj EOs. Results are expressed as mean area percentage (%) ± standard deviation (S.D.) of three independent determinations in triplicate (n = 3).

Compound	*V. officinalis*	*V. jatamansi*	*N. jatamansi*	KI ^a^	Identification ^b^
2-Acetylfuran	-	0.50 ± 0.03		912	1,2
Tricyclene	0.14 ± 0.01	-	t	926	1,2
α-Thujene	0.03 ± 0.00	-	0.06 ± 0.00	930	1,2
α-Pinene	3.39 ± 0.15	0.20 ± 0.01	1.59 ± 0.05	939	1,2,3
3-methyl-Valeric acid	-	0.09 ± 0.00	-	945	1,2
α-Fenchene	-	-	0.01 ± 0.00	952	1,2
Camphene	13.85 ± 0.88	0.29 ± 0.01	0.03 ± 0.00	954	1,2,3
Sabinene	0.16 ± 0.01	-	-	975	1,2
β-Thujene	-	-	0.03 ± 0.00	976	1,2
β-Pinene	2.76 ± 0.12	-	2.73 ± 0.13	979	1,2,3
α-Phellandrene	-	-	0.01 ± 0.00	1002	1,2
α-Terpinene	0.02 ± 0.00	-	0.04 ± 0.00	1017	1,2
p-Cymene	0.32 ± 0.01	0.11 ± 0.01	0.08 ±0.00	1023	1,2
Limonene	1.50 ± 0.04	0.07 ± 0.00	0.21 ± 0.01	1029	1,2,3
1,8-Cineole	0.02 ± 0.00	0.02 ± 0.00	0.18 ± 0.01	1031	1,2,3
γ-Terpinene	0.15 ± 0.01	-	0.07 ± 0.00	1059	1,2
Terpinolene	0.05 ± 0.00	-	0.05 ± 0.00	1088	1,2
2-Nonanone	-		0.01 ± 0.00	1090	1,2
Isoamylvalerate	-	-	0.01 ± 0.00	1081	1,2
Linalool	-	-	0.02 ± 0.00	1096	1,2,3
Fenchol	-	-	0.01 ± 0.00	1116	1,2
Camphor	0.07 ± 0.00	-	-	1146	1,2
Borneol	0.87 ± 0.03	0.17 ± 0.01	0.02 ± 0.00	1169	1,2,3
cis-3-Pinanone	-	-	0.03 ± 0.00	1174	1,2
Terpinen-4-ol	0.18 ± 0.01	-	0.14 ± 0.01	1177	1,2,3
α-Terpineol	0.13 ± 0.01	-	0.15 ± 0.01	1188	1,2
Myrtenol	0.28 ± 0.02	-	0.09 ± 0.00	1195	1,2
Citronellol	0.61 ± 0.03	-	-	1225	1,2,3
Thymol, methyl ether	0.30 ± 0.01	0.05 ± 0.00	0.11 ± 0.01	1235	1,2
Carvacrol, methyl ether	-	-	0.36 ± 0.02	1244	1,2
Furfurylvalerate	-	0.14 ± 0.01		1271	1,2
Bornyl acetate	46.90 ± 1.24	0.42 ± 0.02	0.02 ± 0.00	1285	1,2,3
Undecan-2-one	-	-	0.69 ± 0.03	1294	1,2
1,2,3-Trimethylindene	-	-	0.06 ± 0.00	1299	1,2
Mirtenyl acetate	3.94 ± 0.22	-	0.40 ± 0.02	1326	1,2
δ-Elemene	-	-	0.19 ± 0.01	1338	1,2
α-Cubebene	-	-	0.09 ± 0.00	1351	1,2
Cyclosativene	-	-	0.03 ± 0.00	1371	1,2
α-Copaene	-	-	1.90 ± 0.14	1376	1,2
β-Patchoulene	-	6.02 ± 0.32	0.90 ± 0.04	1380	1,2
β-Cubebene	-	-	0.35 ± 0.02	1386	1,2
β-Elemene	1.30 ± 0.05	0.55 ± 0.02	1.54 ± 0.06	1389	1,2
Cyperene	-		3.42 ± 0.24	1398	1,2
β-Longipinene	-	1.11 ± 0.04	-	1400	1,2
α-Gurjunene	1.13 ± 0.04	-	-	1409	1,2
β-Maaliene	-	-	2.07 ± 0.18	1415	1,2
α-Santalene	-	3.35 ± 0.18	-	1417	1,2
9-Aristolene	-	-	1.42 ± 0.05	1418	1,2
(E)-β-Caryophyllene	0.30 ± 0.02	0.37 ± 0.02	2.94 ± 0.22	1419	1,2,3
Calarene	0.17 ± 0.01	10.57 ± 0.05	8.22 ± 0.44	1433	1,2
α-trans-Bergamotene	-	0.93 ± 0.04	-	1434	1,2
α-Guaiene	-	2.58 ± 0.14	0.05 ± 0.00	1439	1,2
Aromadendrene	-	6.43 ± 0.35	4.17 ± 0.26	1441	1,2,3
Seychellene	-	4.27 ± 0.28	6.90 ± 0.38	1446	1,2
α-Humulene	0.32 ± 0.01	1.29 ± 0.05	0.82 ± 0.04	1453	1,2,3
α-Patchoulene	-	3.35 ± 0.16	0.51 ± 0.02	1456	1,2
*allo*-Aromadendrene	1.21 ± 0.04	-	-	1458	1,2
γ-Gurjunene	-	11.88 ± 0.48	6.36 ± 0.25	1477	1,2
ar-Curcumene	-	-	1.07 ± 0.03	1479	1,2
Germacrene D	0.05 ± 0.00	-	0.50 ± 0.02	1481	1,2
(E)-β-Ionone	0.14 ± 0.01	-	0.19 ± 0.01	1488	1,2
β-Selinene	-	-	2.55 ± 0.11	1490	1,2
Valencene	-	0.19 ± 0.01	8.05 ± 0.37	1496	1,2
Bicyclogermacrene	0.55 ± 0.02	-	-	1500	1,2
γ-Patchoulene	-	0.76 ± 0.03	-	1502	1,2
α-Bulnesene	-	-	0.26 ± 0.02	1510	1,2
γ-Cadinene	-	-	0.07 ± 0.00	1513	1,2
δ-Guaiene	-	8.53 ± 0.24	-	1516	1,2
α-Maaliene	-	-	1.27 ± 0.06	1522	1,2
δ-Cadinene	-	0.07 ± 0.00	-	1523	1,2
Zonarene	-	-	1.77 ± 0.07	1529	1,2
Kessane	-	2.51 ± 0.12	-	1530	1,2
α-Cadinene	0.50 ± 0.03	0.35 ±0.01	0.86 ± 0.04	1538	1,2
Elemol	-	1.08 ± 0.03	-	1549	1,2
Norpatchoulenol	-	8.06 ± 0.25	-	1555	1,2
GermacreneB	0.31 ± 0.01	-	-	1561	1,2
Maaliol	-	17.43 ± 0.56	-	1567	1,2
α-Vatirenene	-	-	3.07 ± 0.21	1570	1,2
Spathulenol	0.84 ± 0.03	1.32 ± 0.04	0.52 ± 0.02	1577	1,2
Spirojatamol	-	-	3.49 ± 0.18	1585	1,2
Globulol	0.84 ± 0.04	0.45 ± 0.02	0.48 ± 0.03	1590	1,2
Viridiflorol	-	0.16 ± 0.01	1.88 ± 0.08	1592	1,2
Guaiol	1.11 ± 0.02	0.57 ± 0.02	-	1600	1,2,3
Ledol	-	-	0.11 ± 0.01	1602	1,2
Hinesol	0.28 ± 0.01	-	-	1641	1,2
*τ*-Muurolol	-	-	1.24 ± 0.10	1642	1,2
Valeranol	0.56 ± 0.02	0.97 ± 0.03	-	1652	1,2
α-Cadinol	-	-	2.24 ± 0.20	1654	1,2
Patchoulol	-	0.41 ± 0.02	-	1658	1,2
Jatamansone	-	-	13.96 ± 0.74	1675	1,2
Valeranal	-	-	4.39 ± 0.25	1706	1,2
Aristolone	-	-	2.44 ± 0.13	1763	1,2
Nootkatone	-	-	0.44 ± 0.02	1806	1,2
trans-Valerenyl acetate	13.18 ± 0.55	-	-	1867	1,2
cis-Valerenylisovalerate	1.53 ± 0.04	-	-	2052	1,2
Total	100.00	100.00	100.00		
Monoterpene hydrocarbons	23.70	0.67	4.83		
Oxygenated monoterpenes	53.29	0.19	0.53		
Sesquiterpene hydrocarbons	4.08	67.23	61.59		
Oxygenated sesquiterpenes	4.44	30.45	23.93		
Others	14.71	1.46	9.123		

^a^ Linear retention index on a HP-5MS column; ^b^ Identification method: 1 = linear retention index; 2 = identification based on the comparison of mass spectra; 3 = Co-injection with standard compounds; t = traces, less than 0.01%.

**Table 3:** Phytochemicals identified in the present study and already elucidated in previous investigations on essential oils of Vo, Vj and Nj. + found; - not found.

Compound	*V. officinalis*	*V. jatamansi*	*N. jatamansi*
α-Thujene	+	-	+
α-Pinene	+	+	+
3-methyl-Valeric acid	-	+	-
Camphene	+	+	-
β-Pinene	+	-	-
p-Cymene	+	+	-
Limonene	+	+	-
1,8-Cineole	+	-	-
γ-Terpinene	+	-	-
Linalool	-	-	+
Borneol	+	+	-
Citronellol	+	-	-
Thymol, methyl ether	-	+	+
Carvacrol, methyl ether	-	-	+
Bornyl acetate	+	+	-
Undecan-2-one	-	-	+
Mirtenyl acetate	+	-	+
δ-Elemene	-	-	+
α-Cubebene	-	-	+
Cyclosativene	-	-	+
α-Copaene	-	-	+
β-Patchoulene	-	+	+
β-Cubebene	-	-	+
β-Elemene	+	+	-
Cyperene	-	-	+
β-Longipinene	-	+	-
α-Gurjunene	+	-	+
β-Maaliene	-	-	+
α-Santalene	-	+	-
9-Aristolene	-	-	+
(E)-β-Caryophyllene	+	+	-
Calarene	-	+	+
α-Guaiene	-	+	+
Aromadendrene	-	+	-
Seychellene	-	+	+
α-Humulene	+	+	+
α-Patchoulene	-	+	-
*allo*-Aromadendrene	+	-	-
γ-Gurjunene	-	+	-
Germacrene D	+	-	-
(E)-β-Ionone	+	-	+
β-Selinene	-	-	+
Valencene	-	+	+
Bicyclogermacrene	+	-	-
γ-Patchoulene	-	+	-
α-Bulnesene	-	-	+
γ-Cadinene	-	-	+
δ-Guaiene	-	+	-
α-Maaliene	-	-	+
δ-Cadinene	-	+	+
Zonarene	-	-	+
Kessane	-	+	-
α-Cadinene	-	+	-
Germacrene B	+	-	-
Maaliol	-	+	-
Spathulenol	+	+	+
Spirojatamol	-	-	+
Globulol	+	-	+
Viridiflorol	-	+	+
Guaiol	+	+	-
Ledol	-	-	+
Hinesol	+	-	-
*τ*-Muurolol	-	-	+
Valerenol	+	-	-
α-Cadinol	-	-	+
Patchoulol	-	+	+
Jatamansone	-	-	+
Valeranal	-	-	+
Aristolone	-	-	+
trans-Valerenyl acetate	+	-	-
cis-Valerenylisovalerate	+	-	-
References	[20,31,32]	[3,15,18,22]	[28,29,30]
